# Therapeutic Promise and Biotechnological Prospects of *Dendroaspis polylepis* Venom Proteins: Mambalgins, Fasciculins, and Dendrotoxins

**DOI:** 10.3390/ijms26209895

**Published:** 2025-10-11

**Authors:** Tomasz Kowalczyk, Martyna Muskała, Janusz Piekarski, Maciej Kowalski, Marek Staszewski, Belma Konuklugil, Patricia Rijo, Przemysław Sitarek

**Affiliations:** 1 Department of Molecular Biotechnology and Genetics, Faculty of Biology and Environmental Protection, University of Lodz, Banacha 12/16, 90-237 Lodz, Poland; tomasz.kowalczyk@biol.uni.lodz.pl; 2 Students Research Group, Department of Medical Biology, Medical University of Lodz, 90-151 Lodz, Poland; martyna.muskala@student.umed.lodz.pl (M.M.); maciej.kowalski1@student.umed.lodz.pl (M.K.); 3 Department of Surgical Oncology, Medical University of Lodz, Pomorska 251, 93-513 Lodz, Poland; 4 Department of Synthesis and Technology of Drugs, Medical University of Lodz, Muszynskiego 1, 90-151 Lodz, Poland; 5 Department of Pharmacognosy, Faculty of Pharmacy, Ankara University, Ankara 06560, Turkey; 6 Department of Pharmacognosy, Faculty of Pharmacy, Lokman Hekim University, Ankara 06510, Turkey; 7 CBIOS-Research Center for Biosciences & Health Technologies, Universidade Lusófona de Humanidades e Tecnologias, 1749-024 Lisbon, Portugal; 8 iMed.ULisboa-Research Institute for Medicines, Faculdade de Farmácia da Universidade de Lisboa, Av. Prof. Gama Pinto, 1649-003 Lisbon, Portugal; 9 Department of Medical Biology, Medical University of Lodz, Muszyńskiego 1, 90-151 Lodz, Poland

**Keywords:** black mamba, mambalgin, dendrotoxin, fasciculin, ASIC1a, Kv1 channels, recombinant toxins, neurotoxins, venom-derived therapeutics, anticancer effect

## Abstract

Animal toxins contain various bioactive peptides and proteins which have evolved to interact in specific ways. As such, they are a good starting point for developing new drugs and vaccines. This paper examines three natural neurotoxins derived from the black mamba (*Dendroaspis polylepis*), which show significant pharmacological potential: mambalgins, fasciculins and dendrotoxins. All three may be of value in the treatment of pain, cancer and neurodegenerative disease. Mambalgins provide similar pain relief to opioids but without the risk of addiction; they act by selectively blocking acid-sensitive ion channels (ASICs), especially ASIC1a. Thanks to this inhibitory activity they also demonstrate selective activity against glioblastoma, melanoma and leukemia cells as innovative anticancer drugs. Fasciculins are very strong inhibitors of acetylcholinesterase (AChE) and hence offer promise in multi-target drugs and as treatments for treating Alzheimer’s disease. Dendrotoxins such as DTX-K and DTX-I are able to modulate neuronal excitability and synaptic transmission by blocking voltage-gated potassium channels (Kv1.1, Kv1.2, Kv1.6); both have been shown to be effective against cancer cells, and to influence the cardiovascular, immune, and digestive systems. Recent advances in recombinant biotechnology and protein engineering have allowed their safe production with increased therapeutic value. The review examines the translational potential of *D. polylepis* venom proteins and highlights the need for additional preclinical research on bioactive molecules of toxin origin.

## 1. Introduction

Paracelsus, regarded as the father of modern toxicology, formulated the principle “*Sola dosis facit venenum*” (“the dose makes the poison”) as early as the 16th century [1,2]. Thus lies the paradox of toxins, which possess both destructive and healing potential [3]. While toxins may be a tool for defense or prey capture in their natural context, they can also be used for treatment in controlled laboratory and clinical conditions. A notable example is the use of conotoxins from cone snails [4,5], which are naturally used to immobilize prey and have inspired the development of painkillers. Captopril, a widely prescribed antihypertensive pharmaceutical agent, was developed based on peptides that enhance the action of bradykinin, a bioactive peptide found in the venom of the South American viper (*Bothrops jararaca*) [6,7].

One of the largest and most dangerous venomous snakes in sub-Saharan Africa is the black mamba (*Dendroaspis polylepis*) [8,9,10]. Its venom is extremely potent and acts rapidly; a single bite can deliver several hundred milligrams, with only a few dozen being enough to kill a human. Proteomic analyses have found the venom to contain forty different proteins and peptides, including mambalgins, fasciculins, and dendrotoxins. However, in addition to their toxic properties, these substances also have potential in research and therapy [11,12].

The discovery of mambalgins, which act on acid-sensitive ASIC ion channels, has opened up new possibilities for pain management. Rather than causing toxic symptoms, mambalgins were found to exhibit analgesic effects comparable to morphine, but without the risk of addiction. This finding represents a captivating illustration of how animal toxins can stimulate the conception of novel classes of pharmaceuticals [13,14]. Fasciculins are small, three-finger toxins that support the accumulation of acetylcholine in synapses by effectively inhibiting acetylcholinesterase activity. They have been used in studies of cholinergic neurotransmission and can be used as new enzyme inhibitors in experimental studies [15,16]. Finally, dendrotoxins belong to the Kunitz-type protease inhibitor family. These toxins primarily block Kv1.x voltage-gated potassium channels, increasing neuronal excitability. Initially studied as a potential cause of intense neuromuscular excitation in victims, dendrotoxins have quickly become invaluable tools in electrophysiology, enabling the mapping of ion channel function [17]. These three different proteins are presented in Table 1.

Modern biotechnology has opened up entirely new possibilities in toxin research. Genetic engineering allows recombinant versions of toxins to be produced indirectly in expression systems by other organisms, enabling their safe production in large quantities without the need for extraction from natural sources. In addition, site-directed mutagenesis can be used to alter natural protein sequences, enhancing their stability or selectivity towards particular molecular targets, and reducing their toxicity. Peptide optimization strategies can also improve pharmacological profiles, and the development of recombinant antitoxins represents a promising alternative to traditional serum therapies [18] and vaccines, avoiding problems related to immunogenicity and limited availability.

Hence, animal toxins such as black mamba venom represent valuable sources of substrates for modern Medicine and Pharmacology. In an era of protein therapy and molecularly targeted drug design, recombinant toxins could become the basis for next-generation drugs ranging from analgesics to neuroprotectives to anticancer agents. The aim of our work is to examine the therapeutic effects and future clinical applications of three proteins selected from *Dendroaspis polylepis* venom.

**Table 1:** Comparison of selected mambalgin, faciculin and denrotoxin. Based on https://www.uniprot.org/ and https://www.rcsb.org/ (accessed on 6 October 2025).

Toxin	Structure/Type	Protein Size	Mechanism of Action	Molecular Target	Refs.
Mambalgin 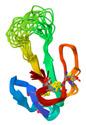 _Mambalgin-1_	Three-finger toxin (3FTx) family peptide	~57 amino acids ~7 kDa	Blocks acid-sensing ion channels (ASICs); produces strong analgesic effects comparable to morphine	ASIC1a and ASIC1b channels, “thumb” domain, electrostatic, hydrophobic interactions, hydrogen bonds	[19]
Amino acid sequence: LKCYQHGKVVTCHRDMKFCYHNTGMPFRNLKLILQGCSSSCSETENNKCCSTDRCNK					
Fasciculin 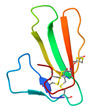 _Fasciculin-1_	Three-finger toxin (3FTx) family peptide	~61 amino acids ~7 kDa	Inhibitor of acetylcholinesterase; prolongs the action of acetylcholine at synapses	AChE enzyme, peripheral anionic site (PAS), hydrogen bonds, hydrophobic, ionic interactions	[15,16]
Amino acid sequence: TMCYSHTTTSRAILTNCGENSCYRKSRRHPPKMVLGRGCGCPPGDDYLEVKCCTSPDKCNY					
Dendrotoxin (I/K) 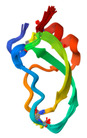 _Dendrotoxin-K_	Kunitz-type peptide (protease inhibitor-like)	~57 amino acids ~7 kDa (6.8 kDa)	Selectively blocks voltage-gated potassium channels (Kv1)	Kv1 potassium channels (Kv1.1, Kv1.2, Kv1.6), electrostatic, hydrophobic interactions	[17]
Amino acid sequence: AAKYCKLPLRIGPCKRKIPSFYYKWKAKQCLPFDYSGCGGNANRFKTIEECRRTCVG					
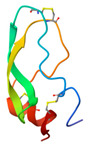 _Dendrotoxin-I_	Kunitz-type peptide (protease inhibitor-like)	~60 amino acids ~7 kDa (6.8 kDa)	Selectively blocks voltage-gated potassium channels (Kv1)	Kv1 potassium channels (Kv1.1, Kv1.2, Kv1.6), electrostatic, hydrophobic interactions	[17]
Amino acid sequence: QPLRKLCILHRNPGRCYQKIPAFYYNQKKKQCEGFTWSGCGGNSNRFKTIEECRRTCIRK					

## 2. Study Design

**Figure 1:**
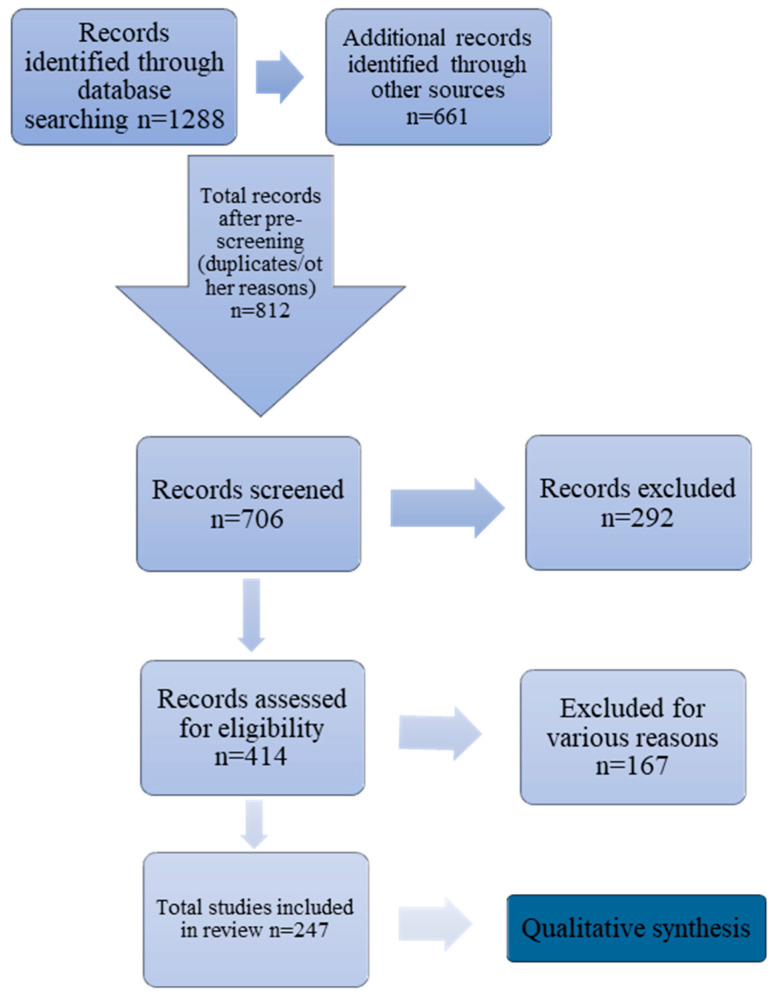
Diagram of the screening procedure.

The literature was reviewed for studies concerning the medical properties of proteins derived from *Dendroaspis polylepis* venom, as well as general information about mambalgins, fasciculins and dendrotoxins. The databases PubMed, Google Scholar and Web of Science were searched using the keywords “*Dendroaspis polylepis*,” “mambalgin,” “fasciculin,” “dendrotoxin,” “anticancer/analgesic/medical properties of venom proteins,” and “recombinant venom toxin.” The initial search period covered the last 25 years, but was expanded to include the 1980s and 1990s when the first studies of black mamba venom proteins were performed. Full-text articles were assessed for eligibility, and some were rejected due to inconsistency with the topic, lack of necessary information, or vagueness regarding inter alia protein origin, study control group information, non-specific mechanism, and for methodology not meeting criteria or language limitations (Figure 1). Additional references were identified by reviewing the reference lists of relevant articles.

## 3. Therapeutic Applications of Animal-Derived Protein Toxins: From Molecular Targets to Clinical Tools

Protein toxins of animal origin represent a distinct class of biologically active compounds that have evolved as specialized tools for predation or defense. These toxins are found in the venoms of various animal species, including inter alia snakes, scorpions and spiders. Many of these toxins display remarkable specificity for molecular targets such as ion channels, membrane receptors and enzymes, which play pivotal roles in physiological processes. Consequently, despite their toxic nature, these compounds have become the focus of intensive pharmacological and clinical research as promising candidates for novel therapeutic agents [20,21,22]. It is important to clarify the distinctions between the terms toxin, venom, and poison, which are often used interchangeably but differ significantly in scientific contexts [23]. A toxin broadly refers to a biologically active molecule, typically a protein or peptide produced by living organisms, that exerts specific, often deleterious effects in another organism at low concentrations [24]. A venom, in contrast, is a complex secretion composed of multiple toxins and other bioactive components. It is synthesized in specialized glands and delivered actively into a target via dedicated apparatus such as fangs, stingers, or spines [25]. Poisons, on the other hand, are toxic substances absorbed passively through ingestion, inhalation, or dermal contact, lacking the active delivery mechanism characteristic of venoms [26].

In animals, it is essential to distinguish between individual toxins (isolated bioactive compounds) and venom, which is a multifaceted mixture of compounds that often act synergistically. This biochemical complexity renders venoms a highly attractive yet challenging source of pharmacologically relevant compounds. Natural toxins are increasingly being used in drug development to inspire the design of both direct preparations and synthetic analogs.

Snake venoms, in particular, have yielded numerous therapeutically relevant proteins such as phospholipase A_2_, metalloproteinases, serine proteases and neurotoxins targeting nicotinic receptors. One of the most well-known is captopril, which is an ACE inhibitor based on the teprotide peptide from *Bothrops jararaca* venom. Captopril revolutionized the treatment of hypertension and heart failure [27,28,29,30]. The groundwork for this discovery was laid in 1949, when it was observed that proteases in Brazilian viper venom triggered vasodilation and blood pressure reduction through the formation of the nanopeptide bradykinin [31].

Another promising group of bioactive peptides, capable of modulating sodium, potassium, and calcium ion channels, are scorpion toxins [32,33]. One notable compound is chlorotoxin, isolated from *Leiurus quinquestriatus*, which binds specifically to glioma cells and is being tested for use in brain tumor imaging and therapy [34]. Scorpion venom has also demonstrated anticancer potential in colorectal and breast cancer cell lines [35], as well as immunomodulatory and antimicrobial activities, suggesting potential as an anti-infective agent [36,37].

Spiders produce a similarly diverse range of venomous peptides. For instance, ω-agatoxin IVA, derived from *Agelenopsis aperta*, blocks P/Q-type calcium channels and serves as a valuable neurophysiological tool [38]. Phα1β, a peptide from *Phoneutria nigriventer*, has shown potent analgesic effects, and have been found to demonstrate greater efficacy than morphine [39,40]. In addition, lycotoxins from spider *Lycosa carolinensis* have also been evaluated for therapeutic use [41,42]. Mollusks, particularly cone snails, produce conotoxins—small peptides with exceptional specificity for ion channels and receptors. A prime example is ziconotide (synthetic ω-conotoxin MVIIA), currently used to manage severe neuropathic pain [43,44] by blocking N-type calcium channels [45,46].

Insects such as bees and wasps also secrete pharmacologically active peptides. Melittin, the major component of bee venom, exhibits cytolytic properties and is under investigation for its anticancer and antimicrobial potential [47,48], and has demonstrated antiproliferative activity in gastric [49], ovarian [50], liver [51], lung [52], and breast cancers [53]. Another bee venom peptide, apamin, selectively blocks potassium channels and has been studied for its neuromodulatory effects [54,55].

Although less frequently highlighted, venom from fish and marine animals may also be involved in experimental studies. A notable example is stonustoxin (SNTX), a heterodimeric protein (~150 kDa) from stonefish (*Synanceia*), which displays potent cytolytic and vasodilatory properties [56]. Moreover, SNTX-derived fragments have shown anticoagulant effects and immunomodulatory potential [57,58,59].

In summary, animal-derived toxins offer multifaceted biological activity and have considerable potential in numerous medical fields including Cardiology, Neurology, Oncology, Immunology, and Dermatology. Their study has already led to the development of several groundbreaking drugs. Ongoing research will likely uncover additional therapeutic applications, paving the way for more precise and personalized treatment strategies based on these naturally evolved molecules [60].

## 4. Biotechnology and Recombinant Proteins in Medicine

Recombinant protein biotechnology is an important tool for engineering protein toxins and creating medical applications for them. Traditionally, such proteins were obtained from natural sources such as animal, bacterial and fungal venom, resulting in limited availability and variable composition, as well as difficulties in purification. However, the introduction of recombinant DNA technology has enabled the production of pure, homogeneous proteins in expression systems such as bacteria (e.g., *Escherichia coli*) [61,62], yeast (e.g., *Pichia pastoris*) [63,64,65], mammalian cells [66], insect cells [67,68], and even plants [69,70,71,72]. Furthermore, large quantities of highly pure proteins can be obtained and further modified to increase safety and efficacy through the use of gene cloning and expression in heterologous systems [73,74,75], for example botulinum toxin type A (BoNT/A). The use of recombinant variants with a shorter duration of action are expected to improve safer without posing a full-toxicity risk [76,77].

Another promising direction is the design of chimeric therapeutic proteins based on combining toxin fragments with antibodies (‘immunotoxins’), which are being investigated for use in cancer therapy [78,79]. Immunotoxins based on diphtheria toxin, for example, consist of the catalytic domain of the toxin linked to an antibody that recognizes a tumor antigen [80,81,82]. The denileukin diftitox IL-2 (interleukin-2) immunotoxin targets cancer cells with the IL-2 receptor, primarily T-cell lymphomas (CTCL) [83,84]. Moxetumomab pasudotox is a *Pseudomonas aeruginosa* (PE38) immunotoxin linked to an anti-CD22 antibody, which is used to treat refractory hairy cell leukemia [85].

While most clinical immunotoxins are based on bacterial or plant toxins, some are based on animal or venomous toxins. These include TM-601, a chlorotoxin derived from scorpion venom (*Leiurus quinquestriatus*) conjugated with iodine-131, which selectively binds to glioma cells [86,87], and contortrostatin from *Agkistrodon contortrix*, which acts on integrins and inhibits angiogenesis and tumor cell adhesion [88,89].

Another important application is the production of antitoxins and vaccines [90]. This area of research, which has a direct impact on everyday life, employs recombinant toxin fragments to act as safe antigens for eliciting an immune response. Examples include monoclonal antibodies and antibody fragments which can neutralize toxins from inter alia snakes, scorpions and spiders, with greater selectivity and reduced risk of adverse effects. This is an alternative to traditional antisera; for example, recombinant antibodies can be created against cobra neurotoxins [91,92,93]. Genetic engineering allows animal and human antibody sequences to be combined to create safer preparations. Recombinant antibodies against lanceolate adder venom (*Bothrops asper*) have already been subjected to clinical trials [94,95].

Due to the complexity of venoms, which contain dozens of proteins with various toxic properties, it is advisable to use a cocktail of recombinant antibodies, particularly for the *Elapidae* family, which can elicit a range of pathophysiological reactions caused by specific components [96]. The use of engineered proteins minimizes the risk of allergic reactions, improves the safety of therapy and provides an alternative to traditional antisera. In addition, recombinant venom peptides, such as dendroxins or mambalgins, produced as recombinant proteins in expression systems represent a potential source of analgesic drugs and tools for neurobiological research.

The development of biotechnological methods and protein engineering enables toxins that were originally a threat to be transformed into innovative therapeutic tools. The ability to introduce structural modifications and control the production process means that recombinant toxins can be produced on a large scale, reproducibly and safely. The perception of these substances has thus shifted from exotic biological curiosities to promising therapeutic tools [97,98,99].

## 5. Characteristics of *Dendroaspis polylepis*

*Dendroaspis polylepis*, commonly known as the black mamba, is considered one of the most dangerous snake species in sub-Saharan Africa due to the extreme potency of its venom. Its natural range extends from Kenya to South Africa, where it inhabits savannahs, shrublands, and riverine forests, typically favoring rocky hillsides. It exhibits both arboreal and terrestrial behaviors, frequently sheltering in termite mounds or hollow trees, and is not currently classified as threatened [100,101,102,103]. The snake itself is not black, with the name referring to the dark bluish-black interior of its mouth, which it displays when threatened; in contrast, other mamba species have paler oral mucosa [101,104]. Its dorsal coloration varies from olive to grayish-brown, with a lighter ventral surface. Juveniles tend to display a greenish-gray hue [100,102,105,106]. The species name *Dendroaspis* translates as ‘tree snake’, while *polylepis* refers to its numerous scales [100].

**Figure 2:**
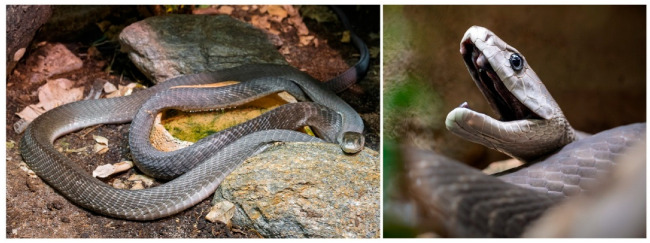
*Dendroaspis polylepis* (Photographer—*Helena Hubáčková*, *Safari Park Dvůr Králové*).

The black mamba is the longest venomous snake in Africa, with adults typically ranging from 2.0 to 2.5 m in length (6.6–8.2 feet), although some specimens exceed 4.3 m (14 feet) [100,102,103,104,106]. It is also among the fastest snakes globally, capable of reaching speeds up to 19 km/h, with anecdotal reports suggesting bursts exceeding 20 km/h [105]. This agility is primarily used for evasion rather than predation. *D. polylepis* is a diurnal and territorial predator that primarily feeds on rodents and other small mammals, birds and occasionally lizards. It employs a strike-and-release hunting strategy, delivering a rapid envenomation followed by retreat until the prey is immobilized [100]. Despite its reclusive nature, the black mamba can display aggressive behavior if escape is not possible. During defensive encounters, it can raise a significant portion of its body, reportedly up to face level in large specimens, and strike repeatedly with high accuracy [103]. Venom yields per bite range from 100 to 400 mg, whereas the estimated lethal dose for an adult human is approximately 10–15 mg [103]. Clinical manifestations of envenomation typically develop within 60 min and include neurological symptoms (e.g., ptosis, paralysis), respiratory depression, and autonomic dysfunction such as profuse sweating [100]. A narrative review analyzing clinical cases of black mamba envenomation reported that 90% of patients received antivenom therapy, with a survival rate of nearly 99%, underscoring the effectiveness of prompt medical intervention [100]. Furthermore, although native to sub-Saharan Africa, *D. polylepis* is occasionally encountered outside its natural range, particularly in Europe and North America, as a result of the exotic pet trade [101]. The snake is depicted in Figure 2.

## 6. Mambalgins

### 6.1. Characteristics of Mambalgins

Mambalgins are peptides composed of 57 amino acids that belong to the three-finger toxin (3FTx) superfamily: an evolutionarily conserved group of non-enzymatic, cysteine-rich proteins commonly found in the venoms of *Elapidae* and *Hydrophiinae* snakes [107,108,109]. The characteristic structure of 3FTx proteins comprises three β-sheet-rich loops (“fingers”) extending from a compact hydrophobic core stabilized by four to five disulfide bridges [108,109]. Based on structural variations within these loops, the 3FTx family is subdivided into short-chain, long-chain, and non-conventional types [108]. Notable members of this family include α-neurotoxins, cytotoxins, and mambalgins [107,108,109]. The discovery of mambalgins dates back to 2012, when Diochot et al. found the venom of *Dendroaspis polylepis* to have potent analgesic properties and no observable respiratory depression: a major limitation of opioid analgesics [14]. The research, conducted at the Institut de Pharmacologie Moléculaire et Cellulaire in France, led to the isolation of two homologous peptides, mambalgin-1 and mambalgin-2, which differ by only a few amino acid residues.

Both homologues were shown to inhibit acid-sensing ion channels (ASICs), a family of proton-gated ion channels involved in nociception [14]. More specifically, mambalgins block ASIC1a in the central nervous system and ASIC1b/ASIC2 in peripheral neurons. Analgesic activity was demonstrated in rodents based on tail-flick and paw-withdrawal tests; however, no such effect was noted in ASIC1a-knockout mice, confirming their channel specificity [14]. Mambalgin use is associated with slower development of analgesic tolerance than opioids. Although mambalgins share certain 3FTx fold structural features with classical neurotoxins like α-bungarotoxin or α-cobratoxin, such as the three-loop architecture, β-sheet content, their size, and stabilization by disulfide bonds, they exhibit low sequence homology (<50%) [107,110]. Mambalgins lack cytotoxic effects and do not interact with nicotinic acetylcholine receptors, unlike typical neurotoxins that induce paralysis [110,111,112].

They primarily exert an analgesic effect through a non-opioid mechanism, i.e., by inhibition of ASICs. Structurally, they are similar to unconventional or weak 3FTx, characterized by an extended second loop and shortened first and third loops [14,110]. Three isoforms have been identified: mambalgin-1, -2 and -3. Mambalgin-2 differs from mambalgin-1 by the replacement of a tyrosine residue (Tyr) with a phenylalanine (Phe) at position 4. Mambalgin-3 also possesses Phe at position 4, with an additional replacement of threonine (Thr) with isoleucine (Ile) at position 23 [113,114]. These minor sequence variations may significantly affect their binding affinities and selectivity toward different ASIC subtypes, influencing their analgesic potency and pharmacokinetics.

While other structurally similar peptides targeting ASICs may exist in black mamba venom, they have not yet been fully isolated or characterized [14,112]. Interestingly, despite the structural similarity of mambalgins to other ASIC-blocking peptides such as PcTx1 (from *Psalmopoeus cambridgei*) or APETx2 (from *Anthopleura elegantissima*), they share no sequence homology, highlighting the diversity of ASIC-targeting mechanisms across venomous species [14,111,112].

### 6.2. Acid-Sensing Ion Channels (ASICS)—Structure, Function, and Biological Significance

Acid-sensing ion channels are proton-gated, voltage-insensitive sodium channels that belong to the degenerin/epithelial sodium channel (DEG/ENaC) family. They are mainly expressed in the central and peripheral nervous systems, but they can also be found in other tissues, such as the heart. These ASICs play crucial roles in many physiological and pathological processes, including pain perception, mechanotransduction, synaptic plasticity, and neuroinflammation. Acid-sensing ion channels were first sequenced in 1996, initiating a new era of research into their structure and function [115,116,117,118,119,120,121]. They are encoded by five homologous genes that undergo alternative splicing, giving rise to multiple isoforms, with the primary human ASIC subunits being ASIC1 (ASIC1a, ASIC1b), ASIC2 (ASIC2a, ASIC2b), ASIC3, ASIC4, and ASIC5 [115,121,122,123,124]. The ‘a’ and ‘b’ isoforms of ASIC1 and ASIC2 differ mainly in the N-terminal portion of the protein [116,121]. The most studied subunits in humans are ASIC1a, ASIC1b, ASIC2a, and ASIC3. In humans, homology between ASIC subunits ranges from 17% to 64% [121].

Each ASIC subunit consists of 500–560 amino acids organized into three main domains: an extracellular domain (ECD), two transmembrane helices (TMD1 and TMD2), and short cytoplasmic N- and C-terminal regions [115,117,118,121]. The ECD, which constitutes about 70% of the total protein mass, is responsible for proton sensing and channel activation. It has a complex structure resembling a hand, composed of the palm, thumb, finger, wrist, and β-ball domains: the palm domain, formed by seven antiparallel β-sheets, serves as the structural scaffold, the finger domain, containing several antiparallel β-sheets stabilized by disulfide bridges, participates in proton sensing, the β-ball domain stabilizes the trimer in the closed state and the thumb domain undergoes conformational changes during channel activation. The ECD is also connected to the transmembrane helices by the wrist domain, which transmits conformational changes initiated by protons binding to residues in the thumb and finger domains (tyrosine, tryptophan, proline, glutamic acid and aspartic acid); these signals are transmitted via the wrist domain to TMD1 and TMD2, resulting in channel opening and Na^+^ influx [115,116,117,118,119,121,122]. The channel remains closed at neutral pH (~7.4) and opens at acidic pH (6.5–5.0). Prolonged exposure to low pH leads to desensitization, preventing excessive activation.

An ion-conducting pore is formed by TMD1 and TMD2. TMD1 stabilizes the channel in the lipid bilayer and interacts with the wrist domain to allow ion passage, while TMD2 lines the pore and regulates gating, forming a hydrophobic barrier in the closed state. The pore loop (P-loop) between TMD1 and TMD2 acts as a selectivity filter. It contains the GxxS motif forming a “GAS strip” (Gly443, Ala444, Ser445 in ASIC1); this serves as a negatively charged environment allowing selective Na^+^ conductance [19,115,117,118,121,122]. The short cytoplasmic N- and C-terminal regions contain regulatory motifs involved in desensitization and interactions with signalling proteins such as PSD-95 and PICK1. Mutations in the N-terminal region can alter ion selectivity, affecting Na^+^ permeability [121].

**Figure 3:**
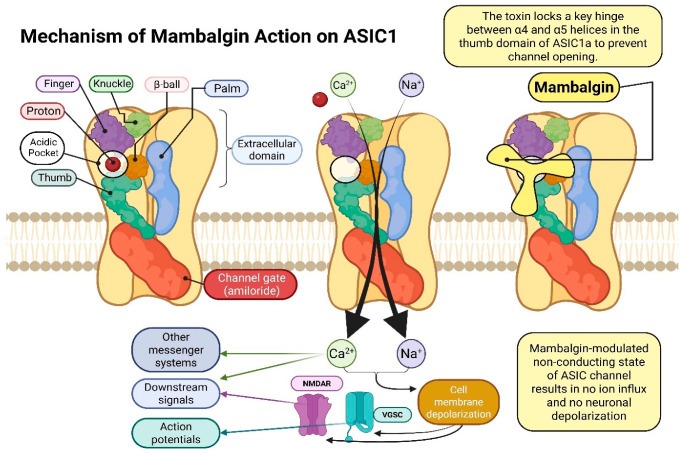
Mechanism of mambalgin action on ASIC1. Created with © 2025 BioRender. Abbreviations: ASIC1—acid sensing ion channel subunit 1; NMDAR—*N*-methyl-D-aspartate receptor; VGSC—Voltage-gated sodium channel.

In summary, ASIC function relies on coordinated interactions between its domains: the finger domain detects protons, the thumb undergoes conformational changes upon binding, the wrist transmits the signal to TMD1 and TMD2, the β-ball stabilizes the closed state, and the transmembrane domains form the Na^+^-selective pore. The cytoplasmic termini modulate desensitization and protein interactions, ensuring proper channel regulation under physiological and pathophysiological conditions [115,116,117,118,119,121,122]. The structure and mechanism of action of these acid- sensing ion channels [125,126,127,128,129,130,131] is shown in Figure 3.

## 7. Genetic Recombination Techniques for the Mambalgin Protein as a Selective ASIC1a Channel Blocker: From Expression to Anticancer Applications

A recombinant form of mambalgin-1 was produced using the PVX (potato virus X) vector [132]. Protein production was achieved in the model plant *Nicotiana benthamiana* [133,134]. The plant codon-optimized PVX-Mambalgin-1 construct was introduced into *Agrobacterium tumefaciens* GV3101 [135] and used to agroinfiltrate *N. benthamiana* leaves [136,137]. Protein expression was confirmed by Western blot and SDS-PAGE, and recombinant protein concentration was quantified using a His-Tag ELISA kit. MTT assays found mambalgin-1 to be cytotoxic against SH-SY5Y neuronal cancer cells but not MCF7 breast cancer cells [138,139]; indeed, proton-gated ASIC1a channels are present in SH-SY5Y neuronal cells but absent in MCF7 cells [132]. The findings demonstrate that the PVX system is an efficient, scalable platform for producing therapeutic peptides, and that plant-based bioreactors are suitable for recombinant protein production, and suggest that modified animal toxins, such as mambalgin-1, could serve as novel anticancer agents.

Recombinant mambalgin-2 [140] has been found to inhibit the proliferation of melanoma cells via ASIC ion channels, specifically the ASIC1a/α-ENaC/γ-ENaC heterocomplex. Acidification of the tumor microenvironment promotes proliferation, migration, and invasiveness of metastatic melanoma cells, with high channel expression associated with poor prognosis [141,142]. The mambalgin-2 protein was produced by cloning into an *E. coli* expression system [143]. Its selective action on ASICs decreased pro-oncogenic factors, including CD44, Frizzled 4, and SNAI phosphorylation, inducing apoptosis in metastatic melanoma cells while sparing Het-1A keratinocytes (EC_50_ ~37 nM vs. ~1000 nM). A mutant mambalgin variant (Leu32Ala), expressed in *E. coli* [144], showed reduced activity against melanoma cells, confirming ASIC1a as the primary target. These findings suggest that recombinant mambalgin-2 represents a promising therapeutic strategy or adjunct to conventional melanoma treatment. These findings underscore the value of genetic engineering for producing large quantities of pure protein for pharmacological research.

Another study investigated the effect of recombinant mambalgin-2 on glioma cells [112]. The peptide selectively blocks the ASIC1a channel, which is highly expressed in U251 MG and A172 glioma cells but absent in normal astrocytes. Given that glioma proliferation and invasion are promoted by an acidic tumor microenvironment, ASIC1a blockade provides a targeted therapeutic strategy. Mambalgin-2 inhibited glioma cell growth and induced apoptosis, as evidenced by reduced cyclin D1 phosphorylation and CDK activity; in addition, the recombinant protein, produced in *E. coli* [140,145,146,147], retained its structural and functional integrity [148]. Functional assays in *Xenopus laevis* oocytes [149] and U251 MG cells demonstrated inhibition of amiloride-sensitive currents with greater specificity than amiloride. Mutant variants with substitutions at Leu32 and Leu34 showed no effect on proliferation, confirming ASIC1a as the molecular target. These findings indicate that selective targeting of ASIC1a with recombinant mambalgin-2 may offer a promising strategy for glioma therapy. Further studies across additional cell lines could inform the development of protein-based therapeutics.

In addition, Bychkov et al. [150] report that mambalgin-2 blockage of the functional ASIC1a channels in the K562 leukemia cell line, which are activated at reduced pH, leads to significant cell cycle arrest in the G0/G1 phase and inhibition of cell proliferation. Molecular studies showed that mambalgin-2 treatment to be associated with reduced expression of cyclins D1 and E1 and their partner kinases CDK2 and CDK4 and increased cyclin inhibitor p21 level. These results confirm that mambalgins may be used not only as potential analgesics but also as innovative anticancer molecules with clinical significance for hemato-oncology patients.

Sudarikova et al. [151] also report that mambalgin-2 exhibited antitumor potential against lung cancer cells in vitro. The peptide selectively bound to ASIC1/α-ENaC/γ-ENaC heterotrimers, resulting in decreased MAPK/ERK and AKT proliferative pathway activity. Consequently, a significant reduction in the in vitro proliferation and migration of A549, metastatic Lewis P29 cells and WI-38 cancer cells was observed, as well as inhibition of tumor growth in a mouse lung adenocarcinoma model. No growth restriction was also observed for normal fibroblasts. These results indicate that mambalgins represent an interesting group of candidates for the development of targeted therapies in lung cancer oncology.

## 8. Fasciculin

### 8.1. Characteristics of Fasciculins

Fasciculins are small, highly toxic proteins belonging to the three-finger toxin (3FTx) family. They represent one of the major components of black mamba venom alongside dendrotoxins (~60%) and other three-finger neurotoxins (~30%), including alpha-neurotoxins and mambalgins [152]. Fasciculins inhibit acetylcholinesterase, leading to excessive accumulation of acetylcholine at neuromuscular junctions, which induces involuntary muscle contractions (fasciculations) thus paralyzing the prey. The name *fasciculin* derives from the Latin *fasciculus* (bundle) and the typical protein suffix-*in*, reflecting their ability to provoke tremors in small bundles of muscle fibers [153,154]. To date, four distinct forms of fasciculin have been identified in mamba venoms: Fasciculin-1 (FAS1), Fasciculin-2 (FAS2, formerly F7 toxin), Fasciculin-3 (FAS3, formerly Toxin C), and Fasciculin-4 (FAS4), with Fasciculin-3 being predominant in black mamba venom. Fasciculin-1 and -2 are composed of 61 amino acids and differ by one to three residues, with molecular weights of 6.765 kDa and 6.735 kDa, respectively [15,16]. Their globular structure is maintained by four disulfide bridges (Cys^3^–Cys^22^, Cys^17^–Cys^39^, Cys^41^–Cys^52^, Cys^53^–Cys^59^), which stabilize the three-finger motif [155,156,157,158]. The protein exhibits a dipolar character, with negatively charged residues concentrated at the C-terminal region and positive charges in the central core [158].

Fasciculins were first isolated in 1983 and their structure was determined by crystallography (PDB 1FAS) at 1.8 Å resolution [153,159]. Unlike classical enzyme inhibitors, they do not directly access the active site of acetylcholinesterase, but rather bind at a peripheral site. Clinically, envenomation by *D. polylepis* is associated with neurological symptoms deriving from the combined action of fasciculins and the other venom components; these include fasciculations, chest tightness, paresthesia, tachypnea and dysarthria [160]. Phylogenetically, fasciculins are structurally related to other α-neurotoxins, such as erabutoxin b, and cardiotoxins, including cardiotoxin VII4, highlighting the conserved nature of the three-finger motif among neurotoxic peptides. Their unique structural and functional properties make fasciculins a valuable model for studying protein–enzyme interactions. They may also form the basis of potential pharmacological applications in Alzheimer’s disease and other conditions associated with cholinergic deficiency; they may be suitable candidates for the design of AChE inhibitor drugs and in research on neuromuscular transmission and synaptic physiology [155,156,157,160].

### 8.2. Mechanism of Fasciculin Action

Fasciculin exerts its neurotoxic properties by inhibiting acetylcholinesterase (AChE), which is responsible for the breakdown of acetylcholine at neuromuscular synapses. Thus, results in the accumulation of acetylcholine in the synapses, causing muscle stiffness, paralysis, and ultimately respiratory failure and death. However, as *Elapidae* snake neurotoxins cannot cross the blood–brain barrier, these effects are restricted to the peripheral nerves. Fasciculins are among the most potent inhibitors in mammals and fish, but their activity is reduced in insects (e.g., *Musca domestica*), reptiles (e.g., cobra), and birds: the acetylcholinesterases of these species lack two conserved peripheral anionic residues which significantly reduces binding affinity. Fasciculin has been tested in vitro on human erythrocytes, rat muscle, guinea pig intestine and uterus, as well as on *Electrophorus electricus* in vivo [16].

The sensitivity of different AChEs to fasciculins varies depending on the tissue and protein concentration (from 10 to 30% inhibition at low concentrations ≤ 0.5 nM to 90–110% inhibition at toxic concentrations). This effect is reversible by atropine [161]. Fasciculin does not directly block the catalytic center of AChE but interacts with its surface by binding to the so-called Peripheral Anionic Site (PAS) in a 1:1 stoichiometry, creating hydrophobic and electrostatic interactions between fasciculin loops and PAS residues. This site is defined by amino acids such as Trp286 and two tyrosines, Tyr72 and Tyr124 [162]. Binding to the PAS is believed to be directed, as indicated by its displacement of the probe propidium, indicating higher affinity. After binding, it prevents access of acetylcholine to the catalytic site, this significantly slowing its degradation without physically blocking the active site [163,164].

Fasciculin has a three-loop structure. Arginine at position 11 (Loop I) is a potential anchor point. Loop II contains five positively charged residues; these are partially compensated by anionic residues in Loop III, which stabilize the complex through electrostatic and steric interactions with AChE. Unlike other toxins, charge compensation occurs between different loops rather than within the same loop. These structural and docking studies have paved the way for experimental verification and toxin/antitoxin engineering [165]. Marchot indicate that fasciculin binding to the PAS of AChE, located at the entrance to the narrow catalytic trench on the surface; the bound enzyme can be visualized as a horseshoe, encompassed by the fasciculin three-finger fold. Penetrating the PAS region, it engages in electrostatic and hydrophobic interactions with amino acid residues, mechanically and electrostatically blocking access to the catalytic trench, preventing acetylcholine from reaching the active site (Ser200-His440-Glu327) [166,167].

Fasciculin slows substrate hydrolysis by restricting diffusion into the trench rather than directly blocking the catalytic triad. Researchers indicate the residues involved in these interactions [160,168]. One is Trp279 (Torpedo AChE numbering), whose chemical modification nearly completely negates fasciculin activity. Another is Trp86, where fasciculin may exert allosteric effects; fasciculin has also been found to be an activator for some substrates in Trp86 → Ala mutants. Mutagenesis analyses also indicate that Trp286, Tyr72, and Tyr124 strongly impact binding, with alterations significantly weakening toxin affinity [169].

When complexed, AChE and fasciculin share a ~2000 Å^2^ contact area [170]; the complex demonstrates high complementarity and a low dissociation constant [171]. The dipole moments of both molecules align, and fasciculin seals the narrow gorge leading to the active site; Loop I points down the outer surface of the gorge, Loop II penetrates the trench, stacking Met33 (fasciculin) against Trp279 (AChE) and Loop III rests in contact with the C-terminal residue [172]. The carboxyl groups of fasciculin do not participate in binding [173]. This configuration, governed by charged residues rather than intermolecular bonds, determines the specificity of fasciculin. Fasciculin 2 mainly acts by altering the active-site conformation in the ternary complex, which can slow proton transfer during enzyme acylation. Therapeutic strategies targeting AChE are assessed according to the allosteric inhibitory role of PAS [174].

Binding has been found to decrease the association (k_12_) and dissociation (k_21_) rate constants of fasciculin by more than three orders of magnitude (from 8 × 10^8^ → 1 × 10^5^ M^−1^ s^−1^ for k_12_, and 750 → 0.4 s^−1^ for k_21_), indicating steric blockade of substrate entry and acylation site exit [163]. Spatial multimedia models by Tai et al. indicate fasciculin binding at the AChE active trench entrance, constricting access and mechanically restricting substrate entry, with this configuration being stabilized by polar and hydrophobic residue interactions. There are two pathways for terminating the catalytic reaction (Thr75) and an alternative (Tyr449) [175].

An in silico and experimental study was performed with the aim of increasing the affinity and specificity of fasciculin for *Torpedo californica* AChE [176]. Of the 13 mutated residues generated by ORBIT (the protein redesign program), five retained the necessary polar contacts. Individually, of these five increased affinity seven-fold. The remaining residues unexpectedly retained them, significantly increasing the cumulative effects. Increased affinity was found to correlate with interaction energy differences rather than total protein energy; this data may be valuable when designing inhibitors via protein and genetic engineering [177].

### 8.3. The Use of Fasciculin in Medical Research

In addition to mambalgin, black mamba venom also contains fasciculin: another protein with medically significant properties associated with its potential neurological effects, and which may also influence pain transmission [178]. It has been proposed to have potential value in the indirect treatment of Alzheimer’s disease [179,180,181,182,183,184,185]. This progressive neurodegenerative disorder, characterized by the loss of nerve cells, is considered a major lifestyle-related disease. In 2021, it is estimated that there were 57 million people worldwide living with dementia, of which 60–70% were cases of Alzheimer’s disease (WHO, 2021) [186]. Physiologically, the disease is associated with deposition of abnormal beta-amyloid and tau protein structures in the brain, along with the loss of cholinergic neurons, leading to the formation of neurofibrillary plaques and tangles. Currently, while some pharmacological approaches targeting reduced acetylcholine synthesis and psychosocial care exist, Alzheimer’s disease remains incurable and only symptomatic and palliative treatment is possible [187,188,189,190,191].

Toiber et al. investigated the role of an alternative acetylcholinesterase (AChE) isoform, N-AChE (N-terminal acetylcholinesterase), in neuronal apoptosis [179]. Using a combination of neural cell lines and molecular and histological analyses, it was found that N-AChE overexpression induces apoptosis via the caspase cascade, i.e., activation of Tau kinase GSK3, Tau hyperphosphorylation, and by altering mitochondrial membrane potential. Fasciculin was observed to act as a specific AChE inhibitor, thus separating the enzymatic function of AChE from its signaling role in apoptosis. It was found N-AChE-S-induced cell death could be prevented primarily through inhibition of GSK3 or caspases, by forced overexpression of anti-apoptotic Bcl2 proteins, and by AChE silencing

These findings support the potential of AChE inhibitor therapy in early-stage Alzheimer’s disease, which is in line with the cholinergic hypothesis. Briefly, by inhibiting the degradation of acetylcholine (ACh), AChE inhibitors increase its availability in the brain, thus compensating for ACh loss due to cholinergic neuron death [192].

Fasciculin not only inhibits the activity of AChE but also modulates its interactions with other molecules, including amyloid-β (Aβ). Hu et al. used it as a fluorescent marker to track AChE transport in neurons exposed to Aβ. It was found that AChE labeled with Fasciculin-2 was transported to intracellular vesicles, primarily late endosomes and early lysosomes, confirmed by the LAMP-1 marker. However, in the presence of exogenous Aβ, accumulation increased and shifted from the perinuclear region to the peripheral cytoplasm. Amyloid-β raises AChE levels by reducing degradation and enzyme release and by disrupting intracellular trafficking, potentially causing enzyme accumulation in abnormal locations. Impaired AChE transport to lysosomes may promote neurodegeneration by inter alia neurotoxin accumulation. As with fasciculin, blocking the enzyme’s active site offers dual therapeutic benefit by simultaneously inhibiting AChE activity and reducing amyloid-β deposition. Substances based on fasciculin may elicit fewer adverse effects than conventional treatments by acting allosterically, rather than by directly blocking the catalytic center [180].

Nachon et al. determined the crystal structure of the hAChE–FAS-2 complex, comprising human acetylcholinesterase (hAChE) in complex with fasciculin II (FAS-2) and a hydroxylated derivative of huprine [181]. It was found that fasciculin bound to PAS, structurally blocking substrate access. FAS-2 served as a reference ligand for studying PAS, thus enabling analysis of dual inhibitors acting on both PAS and the catalytic site. This can facilitate the development of multitarget-directed ligands (MTDLs) that function as AChE inhibitors and Aβ aggregation blockers [193,194].

An in silico study evaluated 15 snake venom toxins, including FAS-2, as potential AChE inhibitors for Alzheimer’s therapy. It was found that by using FAS-2 as a model, it was possible to identify ligands with similar binding potential [182].

A study of embryonic mouse AChE found it to be potentially analogous to pathological AChE in Alzheimer’s disease, due to it showing altered PAS and reduced binding affinity. Two molecular forms appear to play a role in mouse brain development: a monomeric (G1) and a tetrameric (G4) form. Of these, the G1 form exhibited lower binding and enzymatic activity, suggesting it may play a role in amyloid plaques and could represent a therapeutic target [183]. Dajas et al. confirmed two types of AChE in rat substantia nigra via fasciculin microinfusion. Fasciculin caused strong, localized AChE inhibition (~87%), without cell death or changes in dopamine, demonstrating that the enzyme functions independently of cholinergic transmission [184].

Anderson et al. report that fasciculin neither affects presynaptic transmitter release nor blocks postsynaptic receptors in mouse diaphragm preparations [185]. Blasina et al. found FAS-II to influence chick retinal development, resulting in significant morphological changes without toxic effects; this supports the hypothesis that PAS inhibitors influence non-cholinergic neurodevelopmental processes [195].

**Figure 4:**
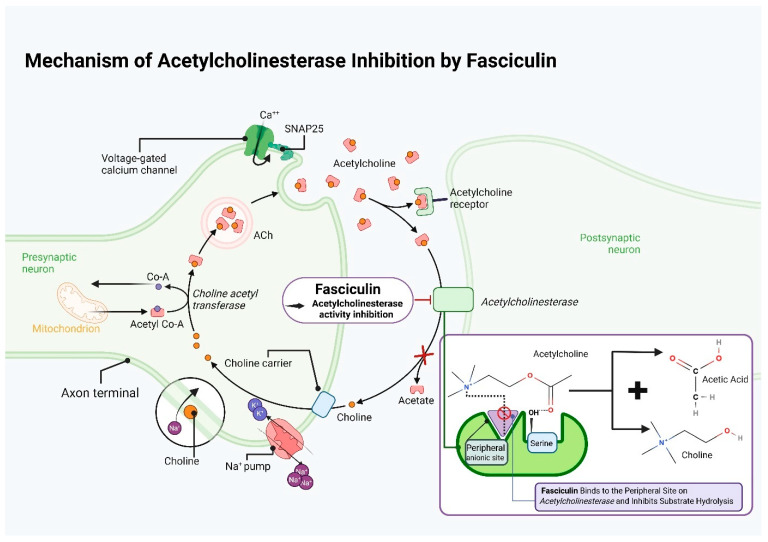
Mechanism of acetylcholinesterase inhibition. Created with https://www.biorender.com/ (accessed on 6 October 2025). Abbreviations: Co-A—Coenzyme A; SNAP25—Synaptosomal-Associated Protein (25 kDa).

Research into fasciculin has been supported through genetic engineering and proteomics. Marchot et al. resolved the crystal structure of mouse AChE-fasciculin II and identified the residues that play a crucial role in the interaction by extensive mutagenesis; the results enabled the design of allosteric AChE inhibitors for Alzheimer’s therapy [196]. Similarly, da Silva Gonçalves et al. used fasciculin as a template to design AChE inhibitors. This resulted in the design of small-molecule analogs that both inhibit AChE and prevent amyloid-β aggregation, and which may represent next-generation Alzheimer’s drugs [197]. The mechanism of action of fasciculin is summarized in Figure 4.

## 9. Dendrotoxin

### 9.1. Characteristics of Dendrotoxins

*Dendroaspis* venom also contains dendrotoxins (originally named C13S2C3) [8,198]. These homologous proteins have been isolated from several mamba species: α-dendrotoxin isoforms (α, β2, γ, and δ) from the eastern green mamba (*Dendroaspis angusticeps*) [199,200], dendrotoxin I and dendrotoxin K from the black mamba (*Dendroaspis polylepis*) [8,201], and Dv14 from the western green mamba (*Dendroaspis viridis*) [200]. Dendrotoxins are potent presynaptic neurotoxins that bind to the nodes of Ranvier in motor neurons and block voltage-gated potassium (K^+^) channels [198]; this blockage prevents proper repolarization of neurons after an action potential, resulting in excessive acetylcholine release at neuromuscular junctions, resulting in muscle hyperexcitability, leading to paralysis and potentially, fatal respiratory failure [201,202].

Despite their high toxicity, dendrotoxins demonstrate high potency and selectivity, and are widely used in neurobiology to study potassium channels and nerve conduction [199,200]. Each mamba species produces a unique set of dendrotoxins with different amino acid sequences and K^+^ channel affinities [199,200]: of these, α-dendrotoxins are the most widely studied and are selective for Kv1.1 and Kv1.2 channels [200], β-dendrotoxins have a different binding profile and affect other Kv channel variants [199], while κ-dendrotoxins are particularly potent against Kv1.2 channels and are primarily found in green mamba venom [200]. Functional classification is based on the specific potassium channels targeted, e.g., dendrotoxin K is selective for Kv1.1, κ-dendrotoxin for Kv1.2 or Kv1.6, while others exhibit a broader spectrum, blocking multiple channel subtypes [198,200].

The ability of dendrotoxins to inhibit repolarization after action potentials by selective blockage of potassium channels makes them invaluable in research into potassium channel physiology, neuronal excitability and synaptic transmission; they are also suitable for modeling neurological disorders related to ion channel dysfunction. Their specificity and high potency allow precise study of neuronal conduction and the role of potassium channels in both normal and pathological conditions [198,200,202].

### 9.2. Structure of Dendrotoxins

Dendrotoxins are small proteins with a mass of approximately 7 kDa, being typically composed of 57–60 amino acids [8,203,204]. They are classified as Kunitz-type protease inhibitors, which act on proteolytic enzymes such as trypsin and chymotrypsin, as well as Alzheimer’s amyloid precursor protein (APP) [205] and tissue factor pathway inhibitor (TFPI). However, the primary function of a dendrotoxin, is not enzyme inhibition but blocking voltage-gated potassium (K^+^) channels [206].

Dendrotoxin structure is stabilized by three disulfide bridges; this arrangement confers resistance to denaturation and proteolysis. These Kunitz domains, comprising two short α-helices and a three-stranded β-sheet, have been utilized as scaffolds for the development of biopharmaceutical drugs [207,208,209]. Dendrotoxins are typified by a compact, spherical shape and the presence of a specific “inhibitor” loop that interacts with the outer part of the potassium channel, often near the outer pore gate. The amino acid residues within this loop determine the selectivity of a dendrotoxin for a particular potassium channel subtype, e.g., DTX-K and DTX-I correspond to Kv1.1 and Kv1.2 [210].

Structurally, a two-turn α-helix is located near the C-terminus, and a short 3_10_-helix near the N-terminus, with the central region occupied by a double-stranded, antiparallel β-sheet, connected by a β-turn (residues 27–39 in DTX-I numbering), which is essential for channel-blocking activity; these features, α-helices, β-sheets and bends are stabilized by abundant lysine and arginine residues [8,17,203,204,211,212,213]. The protein conformation is kept rigid and stable by three pairs of disulfide bridges (-S-S-), which confer resistance to degradation, high temperature and enzymatic digestion. In DTX-I, the cysteine residues form obligatory bridges at C7-C57, C16-C40, and C32-C53 [8,17,211,212].

Dendrotoxins are basic, with a high concentration of positively charged residues forming cationic domains critical for potassium channel binding. For instance, dendrotoxin I contains seven arginines, seven lysines, and three glutamates, with clusters of positive charges in the β-turn region (Lys26, Lys27, Lys28), near the C-terminus (Arg52, Arg53, Arg54, Lys57), and near the N-terminus (Arg2, Arg3, Lys3). These residues play a major role in binding to potassium channels and determining channel selectivity [210].

### 9.3. Mechanism of Dendrotoxin Action

Dendrotoxins act through the selective and reversible blockade of voltage-gated potassium channels from the Kv1 family, primarily Kv1.1, Kv1.2, and Kv1.6, located on the presynaptic membrane of neurons, especially at neuromuscular junctions and near the nodes of Ranvier. Dendrotoxins bind to the external part of the channel pore, stabilizing its closed state and preventing the efflux of K^+^ ions during the repolarization phase of the action potential, thereby prolonging the action potential. This leads to extended depolarization of the presynaptic membrane, suppressing the fast potassium current (f_1_), and prolonging the opening of voltage-gated calcium channels (Ca^2+^), resulting in excessive Ca^2+^ influx into the nerve terminal. Elevated intracellular Ca^2+^ levels trigger an increased release of the neurotransmitter acetylcholine (ACh) into the synaptic cleft, causing prolonged muscle excitation, followed by muscle fatigue and paralysis. Electrophysiological recordings show increased amplitude and frequency of terminal potentials. The toxic effects arise from hyperstimulation of the neuromuscular system, manifesting as tremors, spasms, and, in severe cases, paralysis due to receptor desensitization or impaired muscle repolarization. Loss of control over respiratory muscles can, if untreated, result in death by asphyxiation. Structural studies indicate that dendrotoxin binds eccentrically to the Kv channel, interacting with the channel turret and two adjacent subunits without fully occluding the pore. This binding is reversible and characterized by high affinity (nanomolar K_e_) and slow dissociation from the channel [198,213,214,215,216,217,218].

### 9.4. The Use of Dendrotoxin-K in Medical Research

Dendrotoxin-K (DTX-K) is a highly selective inhibitor of Kv1.1 potassium channels. It has been widely used as a precise research tool in neurophysiology, oncology, cardiology, and gastrointestinal studies. Its mechanism involves blocking K^+^ efflux, leading to prolonged membrane depolarization, modulation of cellular excitability, and alterations in synaptic transmission [215].

In Oncology research, DTX-K has been shown to inhibit tumor cell proliferation. Jeon et al. demonstrated that Kv1.1 channel blockade in gefitinib-resistant H460 cells significantly reduced cell survival and caused G1 phase cell cycle arrest, indicating inhibition of the G1/S transition. This effect was confirmed in vivo using xenograft models in nude mice, where direct intratumoral injection of DTX-K significantly reduced tumor growth [219]. Jang et al. also confirmed the medical usefulness of DTX-K, observing a 72–84% reduction in tumor growth in A549 xenografts after 72 h of treatment. Molecular analysis revealed increased expression of cyclin-dependent kinase inhibitors (p21, p27, p15) and decreased cyclin D3 levels, confirming G1/S cell cycle blockade. In addition to its anti-tumor effects, DTX-K exhibits immunomodulatory properties [220]. Fellerhoff-Losch et al. [221] showed that selective Kv1.1 blockade in CD4^+^ T lymphocytes induces strong TNF-α secretion independent of classical TCR/CD3 activation, without affecting IFN-γ, IL-4, or IL-10 levels. This effect involves activation of the non-canonical NF-κB pathway, suggesting potential applications in cancer immunotherapy and chronic inflammatory conditions. In the nervous system, DTX-K influences GABAergic transmission in preganglionic neurons of the paraventricular nucleus (PVN), modulating nitric oxide (NO) signaling via the cGMP/PKG pathway [222]. This mechanism may play a role in regulating blood pressure, heart rate, and sympathetic overactivity. In the gastrointestinal system, DTX-K enhances intestinal peristalsis by stimulating acetylcholine and tachykinin release from enteric neurons [223,224]. In the interstitial cells of Cajal (ICC), Kv1.1 blockade modulates slow waves and rhythmic contractile activity [225].

DTX-K also has relevance in neurological and pain studies. Finnegan et al. [226] demonstrated that DTX-K blocks GABA inhibition by μ-opioid receptors in the amygdala, suggesting potential modulation of analgesic effects and emotional processes. In sensory neurons, DTX-K increases the number of action potentials and lowers the excitability threshold, highlighting its potential in developing novel analgesics for neuropathic pain [227,228]. Furthermore, in atrial myocytes, DTX-K prolongs action potentials, indicating that Kv1.1 may have a role in cardiac electrophysiology and potential impact on arrhythmias [229]. In pancreatic β-cells, DTX-K enhances insulin secretion in mice, suggesting possible applications in diabetes therapy [230].

In summary, dendrotoxin-K is a highly selective Kv1.1 potassium channel blocker that acts at both cellular and systemic levels, modulating tumor cell proliferation, immune function, neuronal and cardiac activity, and gastrointestinal motility. These properties make it not only a valuable research tool but also a promising starting point for developing new therapeutic strategies in oncology, immunotherapy, neurology, cardiology, and gastroenterology.

### 9.5. The Use of Dendrotoxin-I in Medical Research

Dendrotoxin-I (DTX-I) also shows potential medical applications, including in cancer research, due to specific inhibition of Kv1.1 channels. Research indicates that Kv1.1 regulation can play a role in understanding the dynamic control of potassium channel expression in glial cells. Increasing intracellular cAMP concentrations were found to significantly accelerate the degradation of Kv1.1 mRNA, thereby reducing Kv1.1 protein levels and the total potassium current in C6 glioma cells [231,232,233,234,235]. Dendrotoxin-I blocks up to 96% of the potassium current in glial cells, shifting the resting membrane potential from −40 mV to −7 mV. This indicates that Kv1.1 plays a key role in maintaining membrane polarization in these cells. Reducing the potassium current may have potential therapeutic applications in glioma by influencing tumor cell excitability and proliferation [131].

Wang et al. [236] report that C6 astrocytoma cells exhibit a delayed rectifier potassium current generated by RBK1 channels of the Kv1 family. Electrophysiological characterization using patch-clamp techniques revealed high sensitivity to specific blockers. One of the most potent inhibitors was found to be DTX-I, with an IC_50_ (half maximal inhibitory concentration) of 9 nM, indicating that RBK1 channels play a key role in K^+^ conduction. Hence, the observed potassium current appears to be directly associated with RBK1 homomer activity. This finding is crucial for understanding the role of K^+^ conductance in regulating membrane potential and excitability, and potentially, cell proliferation in glial tumors. Hashimoto examined serotonin (5-HT) neurons in the dorsal raphe nucleus (DRN) of control, stress-resistant, and “learned helplessness” (LH) male Sprague Dawley rats [237]. In the LH group, depolarization-induced firing was significantly reduced, despite normal subthreshold properties; however, this firing was restored to control levels by administration of DTX-I, which blocks Kv1.1, Kv1.2, and Kv1.6 channels. In another study, 5-HT neuronal activity was also restored following inhibition of these channels by ketamine treatment, indicating a potential therapeutic target. Pharmacological inhibition of dendrotoxin-I-sensitive Kv1 channels may thus help normalize serotonergic activity in affective disorders [238].

Treatment with DTX-I can also influence the nervous system and certain sensory organs, such as the auditory system. A study of three distinct potassium currents in rat ventral cochlear nucleus neurons by Rothman and Manis found that selective blockage of Kv1.1 and Kv1.2 channels by DTX-I (10–100 nM) facilitated functional differentiation of current types [239]. Similarly, Hamlet et al. demonstrated in chick nucleus laminaris neurons that DTX-I-mediated blockade of Kv1.1 and Kv1.2 alters postsynaptic potential characteristics and the frequency of spontaneous inhibitory potentials; this may have implications for treating disorders associated with abnormal neuronal electrical activity [240].

**Figure 5:**
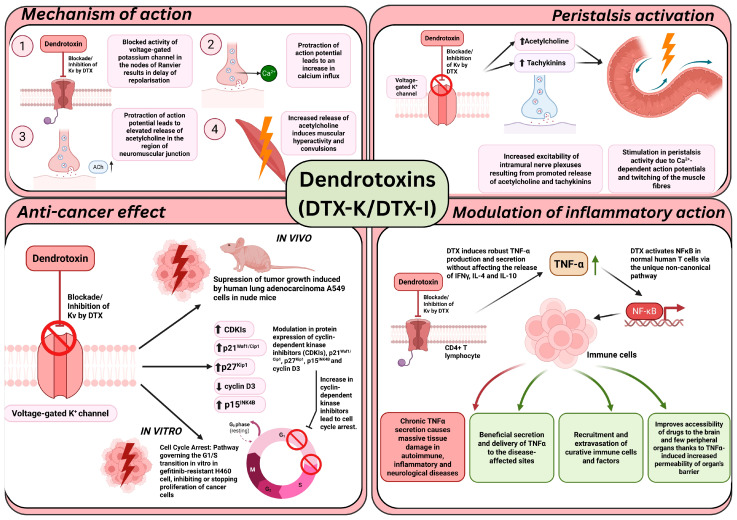
Mechanism of dendotoxin action. Created with © 2025 BioRender. Abbreviations: DTX—Dendrotoxin; Kv—Voltage-gated potassium channel; ACh—Acetylcholine; A549—Adenocarcinomic human alveolar basal epithelial cells; CDKI—Cyclin-dependent kinase inhibitor protein; p21^Waf1/Cip1^—cyclin-dependent kinase inhibitor 1; p27^Kip1^—Cyclin-dependent kinase inhibitor 1B; p15^INK4b^—Cyclin-dependent kinase 4 inhibitor B; H460—human large cell lung carcinoma cell line; TNF-α—Tumor necrosis factor; CD4+—T helper cells; NF-κB—Nuclear factor kappa-light-chain-enhancer of activated B cells.

The cardiac effects of DTX-I were investigated in rat ventricular fibroblasts [241]. Patch-clamp recordings showed that DTX-I blocks depolarization-activated potassium currents; this may prove of value in the development of antiarrhythmic therapies and interventions aimed at limiting pathological cardiac remodeling [242]. Vanzolini et al. [243] note that DTX-I also interacts with acetylcholinesterase (AChE), stabilizing its activity in vitro. This suggests potential applications in diseases with impaired cholinergic signaling, such as Alzheimer’s disease or myasthenia gravis. Finally, DTX-I was also found to block the Kv1.1 and Kv1.2 channels in cerebellar Purkinje neurons [244,245], modulating repetitive firing without substantially prolonging single action potentials. These findings highlight the importance of DTX-sensitive potassium channels in limiting hyperexcitability and may aid in developing therapies for cerebellar disorders such as ataxia. The mechanism of action of dendroxins is summarized in Figure 5.

## 10. Conclusions, Limitations and Future Perspectives

The toxin proteins derived from *Dendroaspis polylepis* venom, such as mambalgins, fasciculins and dendrotoxins, represent a new generation of bioactive molecules with high preclinical potential. All are selective modulators of physiological and pathological pathways, and have been found to interact with particular molecular targets, such as ion channels (ASIC1a, Kv1.1–1.6) and enzymes (acetylcholinesterase).

Mambalgins are known for their powerful pain-relieving properties, which come from their ability to block ASIC1a channels; however, unlike opioids, they are not associated with unwanted side effects. In addition, they appear to have potential application in the treatment of gliomas, melanoma, leukemia, and lung cancer due to their ability to target cancer cells in an acidic microenvironment. Fasciculins are effective AChE inhibitors used in Alzheimer’s disease research and may have a role in multidirectional drugs. Dendrotoxins are able to influence potassium channels, making them candidates for treating cancer, neurological diseases, cardiovascular and gastrointestinal diseases, as well as immunotherapy.

However, there are significant limitations to using animal toxins in therapy. Their immunogenicity and potential toxicity are concerns, as is their lack of bioavailability and stability under physiological conditions. In addition, most of the data originates from preclinical in vitro and animal studies, which restricts their direct clinical application. While the design of recombinant alternatives is developing rapidly, problems still exist with post-translational modifications in heterologous expression systems.

It is worth emphasizing that the development of venom gland organoid technology opens new perspectives in toxin research. Venom-gland organoids provide an ethical, reproducible in vitro platform for producing biologically active venom components and for detailed functional and single-cell studies. This approach complements traditional isolation methods and recombinant techniques, enabling more controlled and reproducible production of bioactive venom proteins. Organoids have already been derived from several species, including, among others, *Naja naja*, *Naja pallida*, *Naja nigricollis*, *Bungarus multicinctus*, *Bothrops jararaca*, *Bothrops atrox*, *Bothrops lanceolatus*, and *Echis coloratus*, and have been analyzed for toxin expression and bioactive peptide production. This technology may provide a safer and more efficient source of research material, thereby facilitating the study of the therapeutic potential and translational applications of snake venom proteins [246,247].

Future research should focus on using protein engineering to optimize the structure of these proteins to increase their stability and selectivity, and to minimize side effects. A promising development is the creation of immunotoxins, which are conjugates of toxins and antibodies. These will allow for the precise delivery of therapeutic molecules to target cells, such as cancer cells. The use of mambalgins and dendrotoxins as components of combination therapies in oncology and neurology is also worth considering. To date, the focus has been primarily on recombinant antibodies neutralizing venom toxins or on using toxin scaffolds to design new binding proteins. However, while the concept of conjugating toxins to antibodies may be an interesting one, it remains in a preliminary phase, and its safety, immunogenicity, and efficacy require further validation. We note this potential in our manuscript, leaving its broader analysis to future, more specialized studies. Furthermore, the scope of translational research should be broadened, and phase I clinical trials aimed at assessing the safety and effectiveness of these molecules in humans should be initiated when possible.

In summary, animal toxins are generally extremely useful in research and treatment, and the proteins from *Dendroaspis polylepis* venom are particularly valuable biomedical tools. Thanks to recent advances in recombinant biotechnology and molecular pharmacology, there is an increasing possibility of converting venom toxins into and effective pharmaceuticals. However, before their therapeutic use can become a reality, it is necessary to overcome certain obstacles, including difficulties in standardizing venom proteins, ethical issues related to obtaining venom from animals, and the high potential production costs, in addition to the short half-life of many toxins and the complexity of approval processes.

## Data Availability

Not applicable.
